# Specific human cytomegalovirus signature detected in NK cell metabolic changes post vaccination

**DOI:** 10.1038/s41541-021-00381-w

**Published:** 2021-09-28

**Authors:** Elena Woods, Vanessa Zaiatz-Bittencourt, Ciaran Bannan, Colm Bergin, David K. Finlay, Matthias Hoffmann, Anthony Brown, Bethany Turner, Shokouh Makvandi-Nejad, Ventzi Vassilev, Stefania Capone, Antonella Folgori, Tomáš Hanke, Eleanor Barnes, Lucy Dorrell, Clair M. Gardiner, PEACHI Consortium

**Affiliations:** 1 School of Biochemistry and Immunology, Trinity Biomedical Sciences Institute, Trinity College, Dublin 2, Ireland; 2 St James’ Hospital, Dublin 8, Ireland; 3 School of Pharmacy, Trinity College, Dublin 2, Ireland; 4 Division of Infectious Diseases and Hospital Epidemiology, Kantonsspital St. Gallen, St. Gallen, Switzerland; 5 Department of Internal Medicine, Division of Infectious Diseases and Hospital Epidemiology, Kantonsspital Olten, Olten, Switzerland; 6 Nuffield Department of Medicine, University of Oxford, Oxford, UK; 7 GlaxoSmithKline Vaccines, Brussels, Belgium; 8 ReiThera s.r.l., Rome, Italy; 9 The Jenner Institute, University of Oxford, Oxford, UK; 10 Joint Research Center for Human Retrovirus Infection, Kumamoto University, Kumamoto, Japan; 11 Oxford NIHR Biomedical Research Centre, Oxford, UK

**Keywords:** Innate immunity, Vaccines

## Abstract

Effective vaccines for human immunodeficiency virus-1 (HIV-1) and hepatitis C virus (HCV) remain a significant challenge for these infectious diseases. Given that the innate immune response is key to controlling the scale and nature of developing adaptive immune responses, targeting natural killer (NK) cells that can promote a T-helper type 1 (Th1)-type immune response through the production of interferon-γ (IFNγ) remains an untapped strategic target for improved vaccination approaches. Here, we investigate metabolic and functional responses of NK cells to simian adenovirus prime and MVA boost vaccination in a cohort of healthy volunteers receiving a dual HCV-HIV-1 vaccine. Early and late timepoints demonstrated metabolic changes that contributed to the sustained proliferation of all NK cells. However, a strong impact of human cytomegalovirus (HCMV) on some metabolic and functional responses in NK cells was observed in HCMV seropositive participants. These changes were not restricted to molecularly defined adaptive NK cells; indeed, canonical NK cells that produced most IFNγ in response to vaccination were equally impacted in individuals with latent HCMV. In summary, NK cells undergo metabolic changes in response to vaccination, and understanding these in the context of HCMV is an important step towards rational vaccine design against a range of human viral pathogens.

## Introduction

Vaccination is the scientific innovation that has arguably had the most positive impact on human health. While it is known that immune events immediately post infection have a profound impact on downstream adaptive immune responses^1,2^, they remain largely unexplored during human vaccination. Understanding early immune engagement is likely to provide new strategies for rational and successful vaccine design, particularly for key pathogens where large-scale vaccine efforts have thus far failed, e.g. human immunodeficiency virus-1 (HIV-1)^3^ and hepatitis C virus (HCV)^4,5^.

Natural killer (NK) cells are innate immune cells that protect against virally infected and transformed cells^6^. However, NK cells can also regulate downstream adaptive immune responses by modulating dendritic cell (DC) activities^7–9^, antibody production^10^ and through the production of large amounts of interferon-γ (IFNγ) cytokine that polarise naive T cells towards a T-helper type 1 (Th1)-type phenotype^11^. Although CD56^bright^ tissue-resident NK cells have recently been described^12^, both CD56^dim^ and CD56^bright^ NK cell subsets are found in the general peripheral blood circulation^13^. CD56^bright^ cells, which account for approximately 10% of circulating NK cells, are potent producers of IFNγ upon stimulation and can also traffic to secondary lymphoid organs such as lymph nodes. The few studies that have investigated the role of NK cells post vaccination in humans have the general caveat of late timepoint samples, more suited for analysis of adaptive immune responses^14–18^. This vaccine study was designed to include early timepoints post vaccination to compare pre- and post-immune activation and metabolism of NK cell subsets in a healthy human cohort in response to a novel dual vaccine against both HCV and HIV-1. This heterologous viral vector vaccine strategy delivers viral antigen by priming with replication-incompetent chimpanzee adenoviruses (ChAdVs) followed by boosting with modified vaccinia Ankara (MVA), which is highly effective at inducing anti-HIV-1 and anti-HCV T cell responses^19^.

Human cytomegalovirus (HCMV) causes a relatively minor illness in humans, but establishes a lifelong latent infection that becomes clinically relevant in immunosuppressed individuals^20^. NK cells are important for immune control of HCMV, and while this has been well defined in particular mouse models^21^, a molecular imprint of HCMV on circulating CD56^dim^ NK cell subsets in humans has also recently been identified^22,23^. These ‘adaptive’ CD56^dim^ cells have altered functional responses including reduced responsiveness to pro-inflammatory cytokines compared to canonical NK cells^24^. We and others have described that cytokines upregulate both glycolysis and oxidative phosphorylation metabolic pathways in NK cells and these are required for key effector functions including IFNγ production and cytotoxicity^25–27^.

This study provided a rare window of opportunity to investigate the direct impact of vaccination on metabolic and functional NK cell responses at both immediate and extended time frames within a human clinical dual-vaccine trial for HIV-1 and HCV. It identified general metabolic changes including increased mitochondrial mass and nutrient receptor expression supporting the sustained proliferation of NK cells in response to vaccination, but also identified a strong HCMV-associated signature that affected both NK cell metabolic and functional responses.

## Results

### Heterologous prime–boost vaccination in healthy individuals causes sustained increases in circulating NK cell numbers

**Fig. 1:**
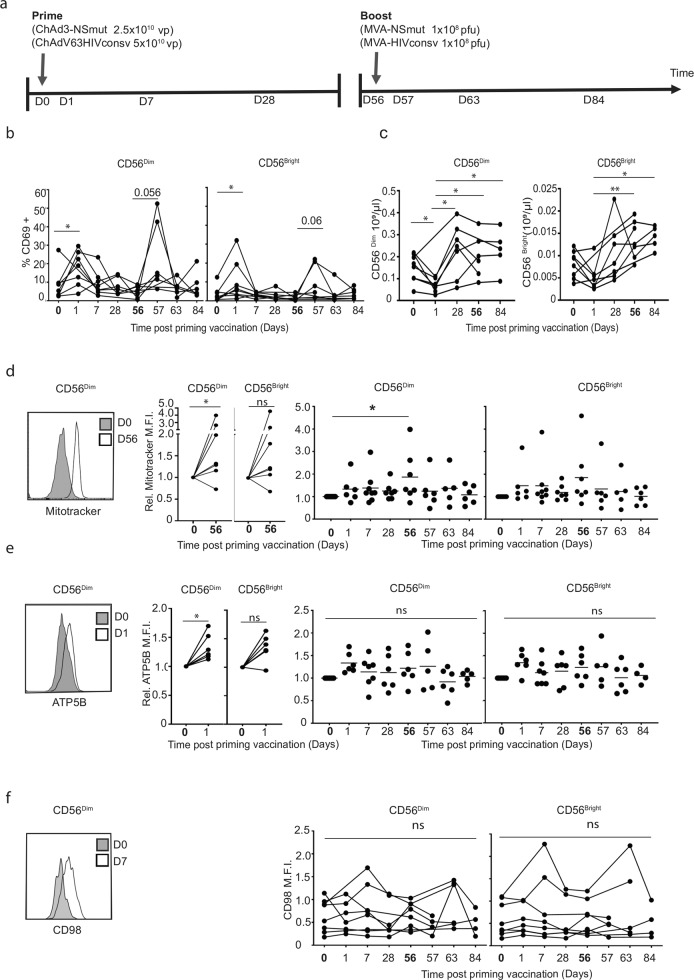
NK cell numbers and mitochondrial mass are increased in response to vaccination. **a** Schematic representation of vaccine schedule and blood draws to assess NK cell responses after vaccination. Arrows at Day 0 and Day 56 indicate the timing of prime and boost vaccines, respectively. Blood samples were taken prior to vaccination and at subsequent timepoints indicated by the Days (D) post-prime vaccine. **b** PBMCs were analysed for % CD69 expression on CD56^dim^ and CD56^bright^ NK cells by flow cytometry at baseline and at indicated timepoints post vaccination (*n* = 7). **c** Absolute peripheral blood NK cell counts before and after vaccination (*n* = 7). **d** Representative histograms, paired donor responses and summaries of relative fluorescence intensity of Mitotracker CMXRos and **e** ATP5B in CD56^dim^ and CD56^bright^ NK cells after vaccination. Data shown are normalised to baseline (Day 0) values (*n* = 6–8 dependent on the timepoint analysed). Paired donor responses are shown for **d** Day 0 and Day 56, **e** Day 0 and Day 1 (*n* = 7). In summary, graphs dots indicate individual donor responses, while lines represent the mean. **f** Representative histograms and summary of mean fluorescence intensity of CD98 in NK cells (*n* = 7). Bold timepoints indicate baseline samples taken on the day of prime (DO) or boost (D56) vaccination. Samples were compared by mixed-model with Bonferroni post hoc test. **c** n.s. not significant, *p < 0.05 and **p < 0.01.

Metabolic changes are crucial for optimal NK cell effector functions; therefore, we investigated the impact of vaccination on NK metabolism in a cohort of healthy human volunteers directly ex vivo after vaccination with a novel heterologous ChAd3-nsmut/MVA-nsmut and ChAdv63.HIVconsv/MVA.HIVconsv vaccine delivered using a ChAdV prime and MVA boost strategy^19,28^. This regimen, summarised in Fig. 1a, has previously been reported to induce long-term (>238 days) polyfunctional anti-HIV-1 and anti-HCV CD4^+^ and CD8^+^ T cell responses^29^, but also early (24 h) innate immune cytokine production^19^. We first confirmed NK cell activation by measuring expression of the CD69 activation antigen (gating strategy, Supplementary Fig. 1a), which was low at baseline on NK cells, but increased 24 h after prime or boost vaccination (Fig. 1b, Days 1 and 57 respectively, *n* = 7), indicating direct in vivo activation of both CD56^bright^ and CD56^dim^ NK cell subsets. This coincided with a transient decrease in total circulating CD56^dim^ NK cell numbers at Day 1 post-ChAdV priming vaccination, which recovered by Day 28 to baseline or above for CD56^dim^ and CD56^bright^ NK cell subsets, respectively (Fig. 1c and Supplementary Fig. S1b).

In line with the emerging concept that NK cells continue to function over extended times post activation^30,31^, a longer-term trend in the increased total circulating CD56^dim^ NK cell numbers was also evident up to Day 84 (*p* = 0.0625) (Fig. 1c), suggesting that heterologous prime-boost may promote the expansion of NK cells in the peripheral circulation. MVA boost but not ChAdV prime also induced an increase in the proportion of less differentiated CD57^–^CD56^dim^ NK cells directly ex vivo, although this effect was transient and limited to Day 57 (Supplementary Fig. 1c). The increased CD56^dim^ NK cells numbers in response to ChAdV vaccination was supported by gradual and sustained increases in mitochondrial mass in the majority of recipients, which reached significance by Day 56, a feature associated with cell activation and increased metabolic activity (Fig. 1d). In contrast, CD56^bright^ NK cells were more variable.

As limited sample availability precluded direct measurement of NK metabolic flux, we used flow cytometry to assess the metabolic profile of NK cells post vaccination. ATP5B, an essential subunit of mitochondrial ATP synthase, was significantly increased in CD56^dim^ NK cells in response to the priming vaccine at Day 1 (Fig. 1e). Nutrient uptake and receptor expression, including the 2-[*N*-(7-nitrobenz-2-oxa-1,3-diazol-4-yl) (2-NBDG) uptake assay (Supplementary Fig. 2a), expression of the system l-amino acid transporter, CD98 (Fig. 1f) and expression of the transferrin receptor CD71 (Supplementary Fig. 2b), were highly variable between donors.

### Metabolic reconfiguration supports heightened NK cell IFNγ production after vaccination

**Fig. 2:**
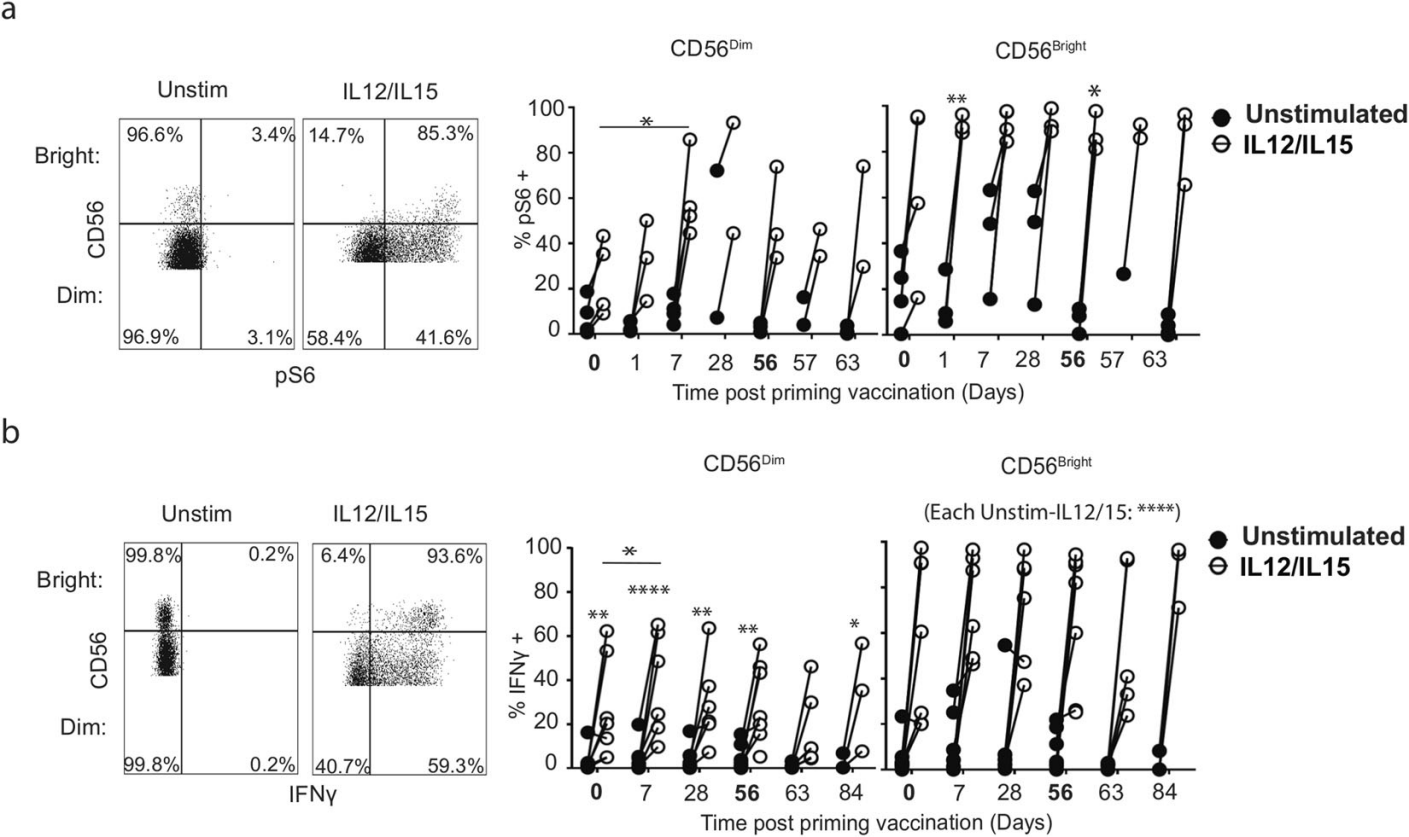
Heightened mTORC1 activity and sustained IFNγ production by CD56^dim^ NK cells after vaccination. **a** Representative flow cytometry plots of NK cells stained with phosphorylated ribosomal protein S6 (pS6)+ or **b** IFNγ+ NK cells at baseline in unstimulated PBMC (left) or after 18 h IL-12 (30 ng) and IL-15 (100 ng) in vitro stimulation (right). Graphs show paired samples of pS6+ or IFNγ+ NK cells at baseline and stimulated at indicated timepoints post vaccination in CD56^dim^ and CD56^bright^ NK cells (*n* = 3–6). Black circles: unstimulated; white circles: IL-12/IL-15. Bold timepoints indicate that baseline samples were taken on the day of prime (DO) or boost (D56) vaccination. **a**, **b** Samples were compared by mixed-model with Bonferroni post hoc test. n.s. not significant. *p < 0.05, **p < 0.01 and ****p < 0.0001.

Cytokine-induced metabolic changes in human and murine NK cells are regulated by mTORC1^25,26^ and mTORC1 is also important for NK cytokine-dependent effector function^32^. To assess mTORC1 signalling, peripheral blood mononuclear cell (PBMC) from ChAdV prime and MVA boost samples were incubated with recombinant interleukin-12 (IL-12) and IL-15, and phosphorylated ribosomal protein S6 (pS6) measured in NK cells by flow cytometry (Fig. 2a). CD56^dim^ NK cell had statistically significantly higher mTORC1 activity in response to cytokine at day 7 post-ChAdV vaccination compared to baseline (Day 0, mean: 25.2%, range: 9–43%; Day 7, mean 59.6%, range: 45–86%; no samples available for later timepoints). At baseline, mTORC1 activity was variable in CD56^bright^ cells, while post-vaccination responses trended towards saturation (Fig. 2a).

IFNγ is a key cytokine produced by activated NK cells that polarises T-cell responses towards Th1-type immunity^11^. To investigate whether NK cell IFNγ production was enhanced by ChAdV priming or MVA boost, PBMCs were stimulated as for Fig. 2a, and IFNγ production by NK cells was measured (Fig. 2b). Responses were variable at baseline; however, IFNγ production by CD56^dim^, but not CD56^bright^, NK cells were significantly increased at Day 7 post priming, suggestive of ‘innate immune training’ of CD56^dim^ NK cells to IL-12/15 stimulation by ChAdV vaccination in vivo^33^. Indeed, previous transcriptomic analysis of whole blood from this cohort revealed induction of IL-12, type I IFN and tumor necrosis factor-α signalling pathways within 24 h of priming vaccine^19^. Induction of heightened mTORC1 activity and IFNγ production had similar kinetics and magnitude in the CD56^dim^ cell subset (Fig. 2a, b), suggesting that the increased capacity to synthesise IFNγ coincided with enhanced mTORC1 signalling at Day 7. In vitro cytokine training experiments supported these data, as while rapamycin did not inhibit overnight stimulation of IFNγ production, it strongly inhibited IFNγ in trained NK cells (see Supplementary Fig. 3). Together, the data show that primary human NK cells are activated in vivo in response to vaccination and suggest changes in NK cell numbers and activation status are supported by increased mitochondrial mass and mTORC1 activity.

### mTORC1 activity and mitochondrial phenotype of CD56^dim^ NK cells after vaccination is influenced by HCMV exposure

**Fig. 3:**
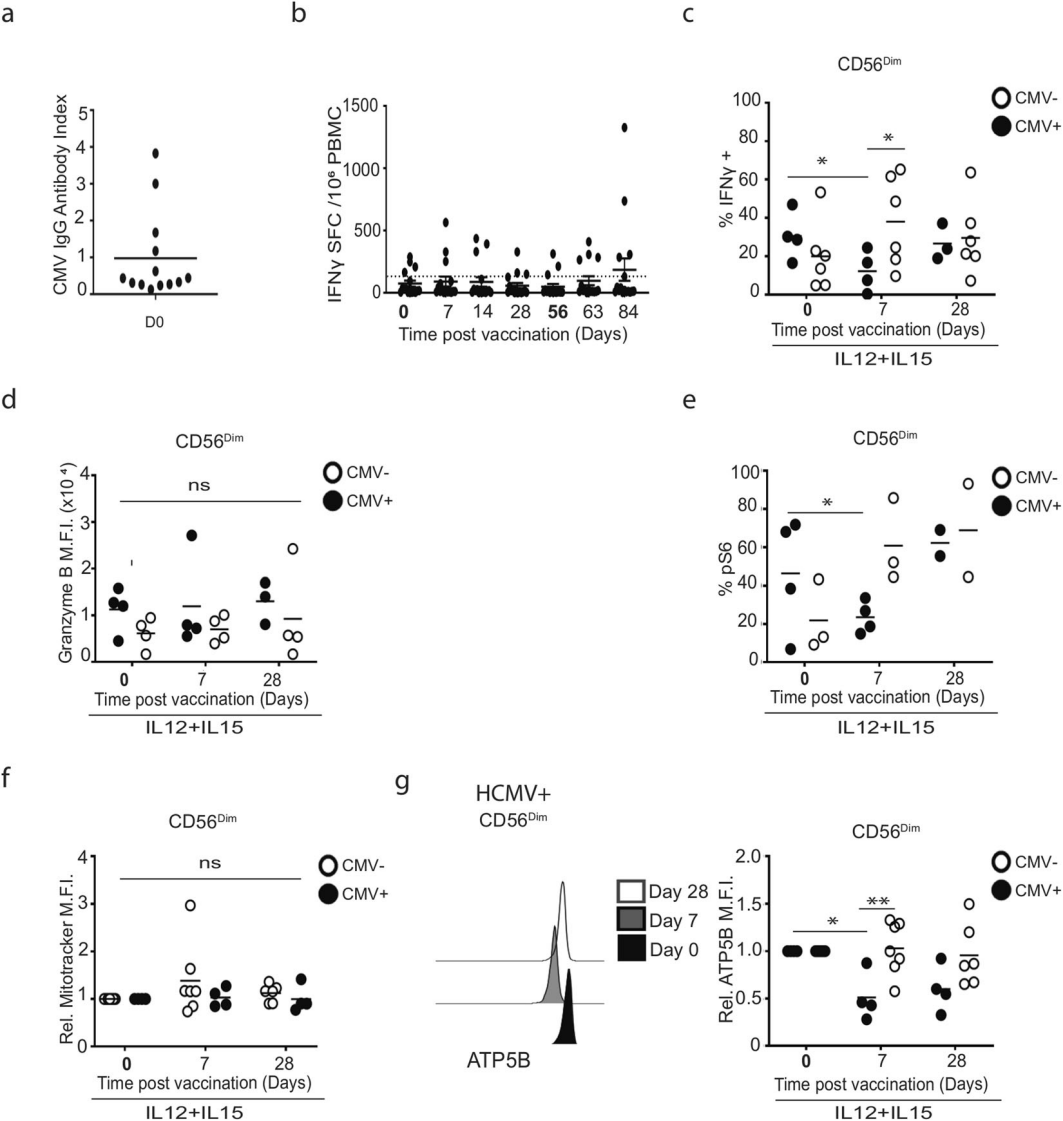
Prior HCMV infection stratifies divergent CD56^dim^ NK cell responses to vaccination. **a** Identification of HCMV seropositive donors in co-vaccinated group. HCMV specific IgG was quantified from pre-vaccination baseline serum by ELISA. The line indicates cut-off for a positive result (*n* = 13). **b** PBMC ex vivo ELISPOT responses to HCMV lysate at baseline and after vaccination. **c**–**e** Stratification of vaccine recipient responses based on HCMV seropositive (black circle) or negative (open circle) status. Each dot shows NK cells stimulated with IL-12/IL-15 overnight and measurement of NK cells expressing **c** IFNγ, **d** granzyme B and **e** pS6 for CD56^dim^ NK cells for pre- and post vaccination (Days 7 and 28). (HCMV−, *n* = 6 for **c**, **d**, *n* = 3 for **e**, HCMV+, *n* = 4). **f** Summary graphs of post-vaccination relative fluorescence intensity of Mitotracker CMXRos and **g** ATP5B in NK cells from HCMV serodiscordant donors normalised to baseline (Day 0) values (*n* = 7 HCMV−, *n* = 4 HCMV+). Representative histogram of ATP5B post vaccination. Samples were compared by two-way ANOVA with Sidek’s post hoc test. n.s. not significant, *p < 0.05 and **p < 0.01.

Given the importance of HCMV in shaping the CD56^dim^ NK cell repertoire, we tested 12 trial participants for prior exposure to HCMV to determine whether HCMV exposure influenced post-ChAdV vaccination responses (samples unavailable post MVA). We identified four HCMV+ donors that tested positive for specific antibodies against HCMV (Fig. 3a) and consistently produced IFNγ in response to HCMV lysate (Fig. 3b). Next, we investigated donors stratified by HCMV serostatus for altered post-vaccination effector responses of CD56^dim^ cells by stimulating PBMCs with IL-12 and IL-15 and measuring IFNγ and granzyme B (GZB) expression. While the pooled data were heterogeneous (Supplementary Fig. 4a), there were striking and significant differences in IFNγ production between HCMV seropositive and seronegative donors post-prime vaccine (Fig. 3c). IFNγ production by NK cells in HCMV− donors was enhanced at Day 7 in CD56^dim^ cells upon cytokine stimulation. In contrast, there was the suppression of the IFNγ response below the pre-vaccination baseline in HCMV+ donors (Fig. 3c). Similar patterns were also observed for levels of IFNγ produced per cell (Supplementary Fig. 4b). Cytokine-driven GZB expression was not affected, suggesting that alterations in IFNγ production were independent of differential responsiveness to IL-12 and IL-15 between HCMV seropositive and seronegative donors after vaccination (Fig. 3d).

As described in Fig. 2, production of IFNγ by NK cells at 1-week post-prime vaccine was temporally associated with increased mTORC1 activation. While pS6 levels were variable in HCMV+ and HCMV− donors at baseline, HCMV− individuals had an increased frequency of pS6 + CD56^dim^ NK cells in response to cytokine activation at 1-week post-prime vaccine (Fig. 3e), while HCMV^+^ individuals had a decreased frequency at the same timepoint, coinciding with the downregulation of NK cell production of IFNγ.

In light of the variability of metabolic responses (Fig. 1), and the importance of OXPHOS in supporting NK IFNγ production^26,34^, we stratified the cohort to investigate if differences in mitochondrial parameters were associated with decreased IFNγ in NK cells in HCMV+ individuals post vaccination. While ChAdV prime vaccine-induced gradual long-term increases in NK cell mitochondrial mass in the overall cohort (Fig. 1d), there was no obvious contribution of HCMV+ upon stratification (Fig. 3f). However, when we quantified ATP5B, its expression in NK cells from HCMV− participants was maintained at baseline levels at Days 7 and 28 post-ChAdV prime in both CD56^dim^ and CD56^bright^ NK cells (Fig. 3g), but was consistently downregulated in HCMV+ participants at these times, suggesting selective downregulation of components of mitochondrial ATP synthase in individuals that had prior exposure to HCMV infection.

### Prior HCMV infection dampens function and metabolism of canonical CD56^dim^, adaptive CD56^dim^ and CD56^bright^ NK cell subsets post vaccination

As HCMV induces expansion of highly differentiated ‘adaptive’ CD56^dim^ NK cells, the alteration in post-vaccination effector responses in HCMV+ individuals could be linked to the expansion of these cells. Adaptive NK cells are phenotypically characterised by high cell surface expression of NKG2C and CD57 and are more recently associated with downregulation of molecules including PLZF- and CD16-associated signalling molecules FcεRγ1 and/or Syk^22,23^.

**Fig. 4:**
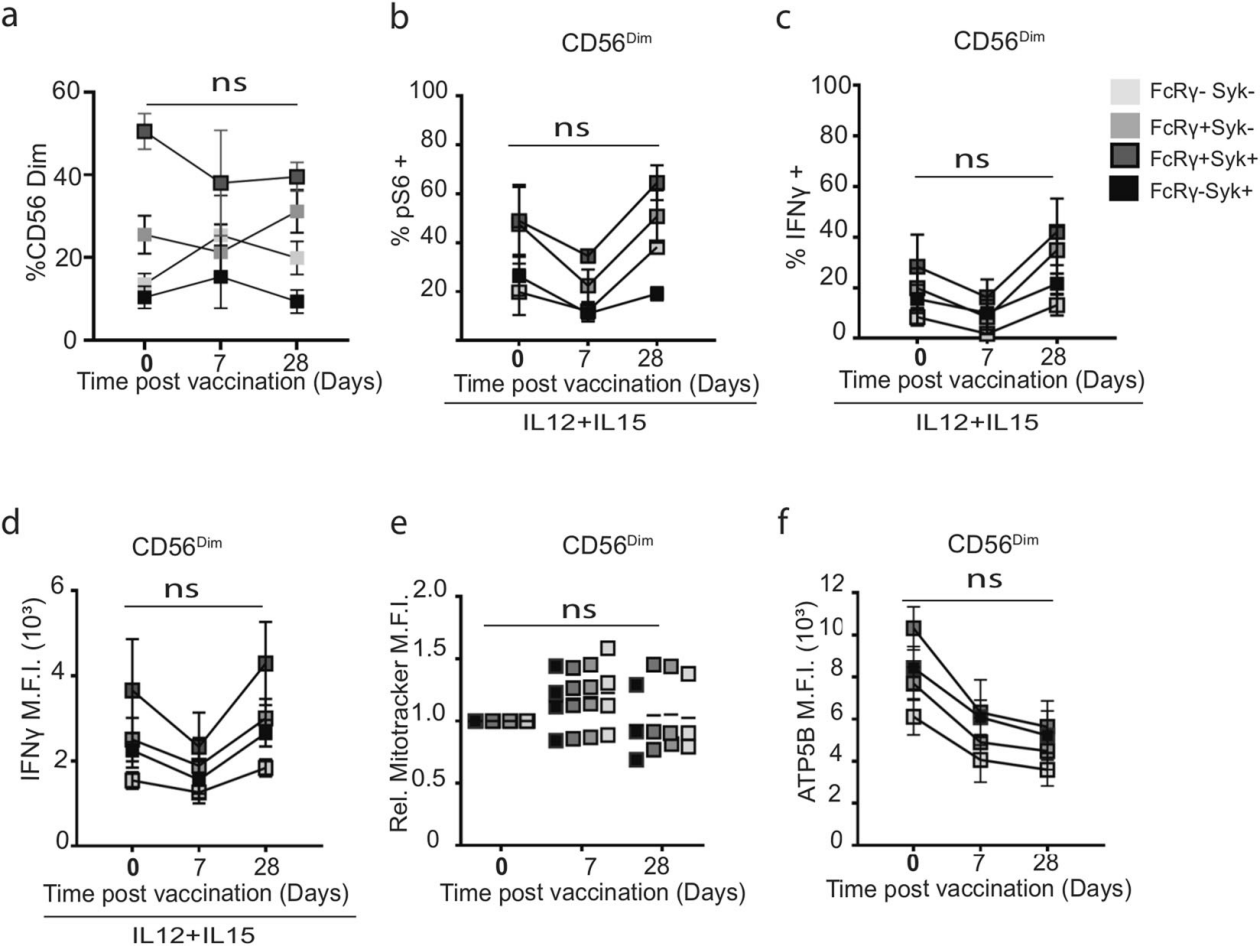
HCMV impacts on functional and metabolic responses of CD56^dim^ NK cell subsets post vaccination. Summary of canonical and adaptive CD56^dim^ NK cells responses at baseline and post-priming vaccination (Day 0, Day 7 and Day 28). Canonical (FcεRγ1+Syk+) or adaptive (FcεRγ1+Syk−, FcεRγ1−Syk+ and FcεRγ1−Syk−) cells were analysed as indicated for **a** ex vivo frequencies of NK subsets, **b** frequency of pS6+, **c** IFNγ+ and **d** IFNγ MFI in response to IL-12/IL-15 stimulation after vaccination (*n* = 4). **e** Summary of post-priming Mitotracker and **f** ATP5B MFI in canonical and adaptive CD56^dim^ NK cells at baseline and Days 7 and 28 (*n* = 4). Error bars show SEM. **a**, **e**, **f** Samples were compared by mixed-model with Bonferroni post hoc test. **b**–**d** Samples were compared by two-way ANOVA with Sidek’s post hoc test. n.s. not significant.

Specific expansion of the FcεRγ1− NK cell subset, with reduced potential mTORC1 signalling, could negatively impact NK cell metabolism and function. To test this, we analysed adaptive NK cell subsets in the vaccinated HCMV+ donors for their frequency and functional responses to cytokine. An expansion of the double-negative (FcεRγ1−Syk−) adaptive NK cell subset was evident in some vaccine recipients, but this was not significant, suggesting that the overall frequency of canonical and adaptive subsets was not altered by vaccination (Fig. 4a).

We next defined the baseline responses of canonical FcεRγ1+Syk+ and adaptive CD56^dim^ NK cells ex vivo (Supplementary Fig. 5) and in response to IL-12/IL-15 activation (Supplementary Fig. 6) in unvaccinated HCMV+ donors, and determined that the canonical FcεRγ1+Syk+ subset were the main producers of IFNγ and exhibited heightened mTORC1 signalling, mitochondrial mass and protein expression in response to cytokine in comparison to adaptive subsets (Supplemental Fig. 6). We then looked at the direct impact of vaccination on these subsets in HCMV+ trial participants. Unexpectedly, we found that rather than a selective inhibition of responses in the canonical NK cell subset, there was a universal trend in decreased mTORC1 activity and IFNγ production in response to IL-12/15 stimulation in all NK cell subsets, including the cytokine-responsive canonical subset (Fig. 4b–d).

In light of this unexpected finding, we next investigated if markers of mitochondrial activity were specifically altered in particular NK cell subsets in HCMV+ individuals post vaccination. While ChAdV prime vaccination-induced long-term increases in NK cell mitochondrial mass in the HCMV− cohort by Day 56 (Fig. 1d), no impact was seen in canonical or adaptive NK cells from HCMV+ up to Day 28 (the last timepoint available) (Fig. 4e). However, as was seen with both mTORC1 activity and IFNγ production, mitochondrial protein ATP5B was downregulated in all HCMV+ vaccine recipients, across all adaptive and canonical NK cell subsets, but this was not statistically significant (Fig. 4f). Together, these data demonstrate that the impact of HCMV+ status on NK cell functional and metabolic responses post vaccination are not explained by the proportions of canonical and adaptive NK cells present.

**Fig. 5:**
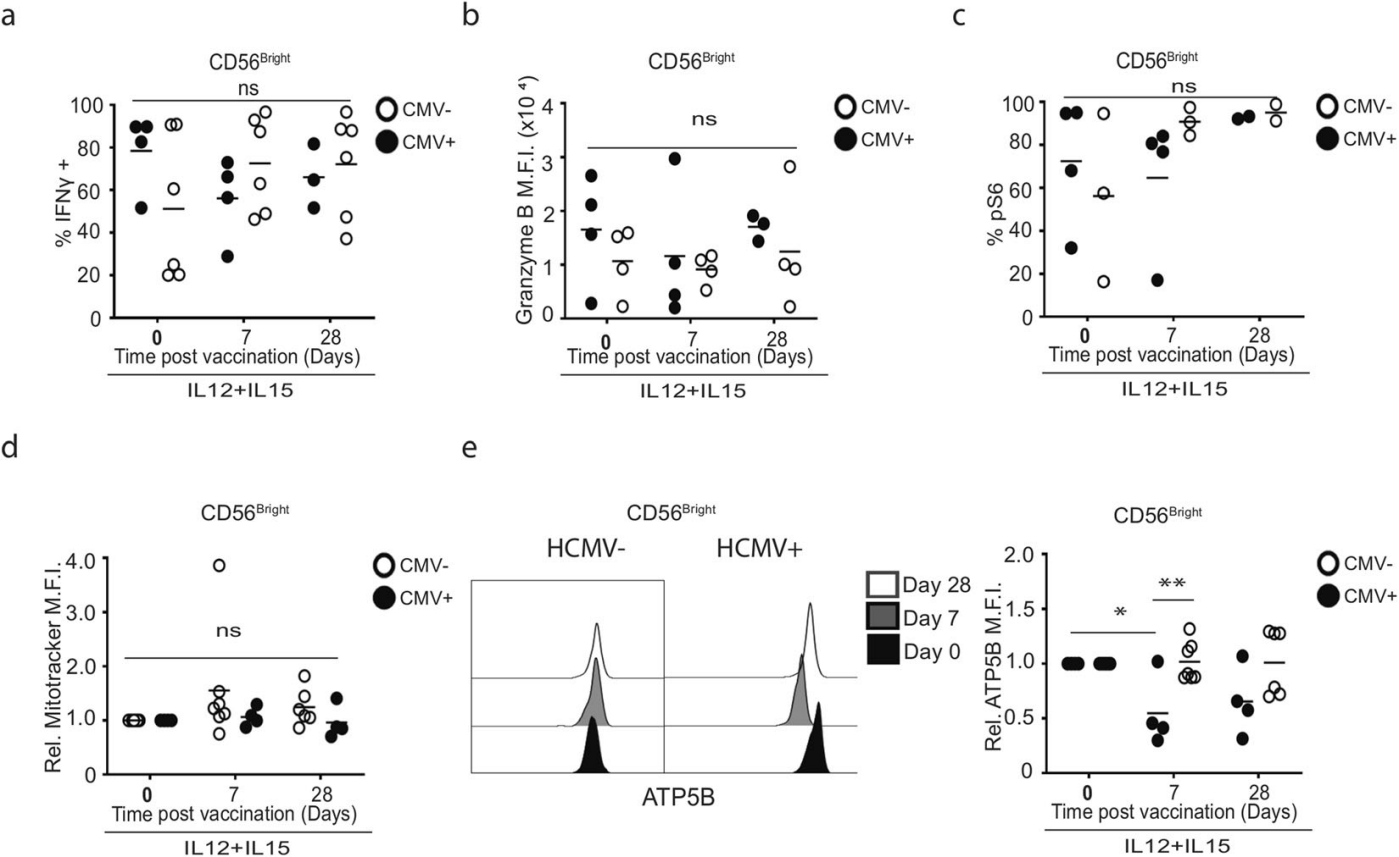
Prior CMV infection impacts ATP synthase expression in CD56^bright^ NK cells in response to vaccination. **a** Summaries of IL-12 (30 ng)/IL-15 (100 ng) stimulated IFNγ+, **b** granzyme B expression and **c** pS6 + CD56^bright^ NK cells in vaccine recipients stratified by HCMV serostatus at pre- and post-vaccination timepoints (Days 7 and 28). (HCMV–, *n* = 6 for **a**, **b**, *n* = 3 for **c**, HCMV+, *n* = 4). Representative histograms and summaries of post-vaccination relative fluorescence intensity of **d** Mitotracker CMXRos and **e** ATP5B in NK cells from HCMV sero-discordant donors normalised to baseline (Day 0) values (*n* = 7 HCMV−, *n* = 4 HCMV+). Samples were compared by two-way ANOVA with Sidek’s post hoc test. n.s. not significant, *p < 0.05, **p < 0.01.

Given the global impact on CD56^dim^ cells, we also investigated if CD56^bright^ cells retained ‘normal’ responses in HCMV+ individuals post vaccination. We observed a similar reduction in pS6, IFNγ production and ATP5B protein expression in CD56^bright^ cells of all HCMV+ donors post vaccination, although only the latter was statistically significant by analysis of variance (Fig. 5). Thus, while a molecular signature of HCMV experienced NK cells can be observed in CD56^dim^ cells of HCMV seropositive donors, the impact of HCMV infection may impact on metabolism and function of all NK cells.

## Discussion

Understanding early immunological events is critical for successful vaccine development and may provide the key to vaccines against HIV-1 and HCV. This is the first study that defines the metabolic changes that NK cells undergo in response to vaccination in healthy human participants. NK cells undergo both short-term and sustained metabolic changes to support their activation, proliferation and immune functions. Latent infection with HCMV had a profound impact on these responses. However, molecular identification of key NK cell subsets determined that this HCMV effect was not exclusively to ‘HCMV experienced’ NK cells, but was observed in all NK cells. Given the global prevalence of HCMV and its endemic nature in developing countries, understanding the impact of HCMV on the immune response to vaccination is a critical research goal.

There is growing evidence that NK cells function over extended time frames after infection, and in parallel with the adaptive immune response. Our data in human volunteers post vaccination support this with acute activation one day after either prime or boost vaccine, and more sustained activation associated with reprogramming of NK cell signalling pathways in terms of IFNγ production (increased at day 7) and proliferation (increased total numbers and mitochondrial mass) at day 56 in response to ChAdV prime vaccination. MVA is reported to induce proliferation in NK cells^35^. Absolute numbers also increased and remained elevated at Day 84 post initial vaccine; however, it is not clear whether this was due solely to the strong ChAdV prime response or if the MVA boost vaccine contributed to this. Notably influenza vaccine also induced a mitochondrial signature containing genes involved in mitochondrial biogenesis and OXPHOS in PBMCs from vaccine responders, suggesting that alterations in mitochondrial metabolism are an important contributing factor to human vaccine responsiveness^36^. Our study clearly identified human peripheral NK cells as a population that undergo profound functional and metabolic changes in response to vaccination. Some of these changes, e.g. increases in mitochondrial mass were identified in NK cells from all vaccinated individuals, while others including mTORC1 activation, ATP5B expression and IFNγ production were largely dictated by prior infection with HCMV. Dissecting these different signatures is key to understanding the early immunological events and mechanisms that will allow strategic targeting of NK cells towards enhanced support of Th1-type immune responses during vaccination. This could also include vaccine delivery systems and adjuvants, e.g. AS01, currently under investigation in malaria and herpes zoster vaccine candidates, caused NK cell activation in lymph nodes with resulting IFNγ production that was important for DC activation and Th1-induced vaccine response^37^.

Early IL-12 production promotes IFNγ secretion by NK cells, which in turn promotes a Th1-type immune response. Transcriptomics data on D1 post-vaccination supports such an early Th1-promoting cytokine signature, which is further supported by an increased ability of NK cells to produce IFNγ in response to cytokine at day 7 post vaccination. Studies have demonstrated T cell-derived IL-2 to be important for a sustained IFNγ response by human NK cells post vaccination^14,18,38^, but we did not find evidence of this in our study. Expression of CD25, the high-affinity IL-2 receptor, was not increased on circulating NK cells and no increases in circulating IL-2 were found at any timepoint measured post vaccination (Supplementary Fig. 7a, b). However, we found a strong mTORC1 signature in NK cells that correlated with IFNγ production a week after vaccination, which suggested IL-2 or IL-15 (which both activate mTORC1 downstream), as key candidates driving this signal. Circulating IL-15 levels were also low (Supplementary Fig. 7c) and it is more likely that draining lymph nodes may represent the site of NK cells interactions where either trans-presentation of IL-15 or local IL-2 production by activated DC or T cells modulate NK cell metabolic and effector responses^39–41^. Supporting this was the transient dip of both CD56^dim^ and CD56^bright^ circulating peripheral NK cell numbers one day after prime vaccine before NK cells recovered with enhanced effector outputs in the peripheral circulation. This transient decrease in peripheral blood NK cells post vaccination has also been observed in other vaccine studies^14,42,43^. The importance of mTORC1 in regulating NK cell effector functions highlights the importance of NK cell flexibility to respond to both earlier and later immune activation events and it is tempting to speculate that initial recruitment of NK cells to lymph nodes, in response to either vaccination or infection, results in IL-15 driving an early metabolic change for sustained NK cell functions. Boost vaccinations, or indeed re-exposure to pathogens, may subsequently drive a rapid antigen-specific IL-2 response, in or from lymph nodes, that in turn would drive a prolonged mTORC1-dependent NK cell activation. Murine conventional NK cells have been reported to circulate through peripheral lymph nodes^44^ and be recruited in response to priming MVA vaccination^42^. Furthermore, it was also recently shown that NK cell migration to lymph nodes was key to control of simian immunodeficiency virus in African green monkeys^45^. However, the mechanisms by which NK cells contribute to and/or regulate the development of protective immunity in response to either natural infection or vaccination are still poorly understood. Indeed, for many vaccination targets, the immune response required to provide natural protection is not known, and correlates for measuring protective T cell immunity are either crude, e.g. ELISPOT or activation-induced markers, or technically challenging and expensive (specific HLA tetramers). In the current study, we found similar ELISPOT responses to HIV/HCV as well as adenoviral hexon peptides between HCMV+ and HCMV− donors (Supplementary Fig. S7d, e), but others have recently reported significantly lower specific T cell responses in HCMV+ compared to HCMV− vaccines for an Ebola vaccine while using a similar ChAdV prime and MVA boost regimen^46^. As the boost MVA vaccine in our study was given at 4 weeks (versus 1 week in the Ebola trial), this could be an important consideration for future trials focused on protective T cell immunity.

It has been known for many years that NK cells are important in immune control of herpesvirus infections, and CMV in particular. The discovery of a molecular signature in human NK cells of prior HCMV infection was a significant breakthrough for the field^22,23^, particularly as previously defined molecular interactions in susceptible mouse strains are not relevant in the context of genetically diverse humans that do not have the key receptor (Ly49H) involved. While correlations of HCMV seropositivity with a high frequency of NKG2C (and CD57) expression in humans serve as good surrogate markers for latent HCMV infection^47–50^, identification of subsets of adaptive NK cells that bear the imprint of HCMV infection has provided a molecular basis for an interrogation of altered functional responses in these adaptive NK cells. In this study, NK cell responses post vaccination (day 7) generally increased (e.g. IFNγ, mTORC1 activity) in HCMV− donors, while the opposite result was observed for HCMV+ donors. It seemed reasonable to assume that adaptive NK cells would account for differences in NK cell responses in HCMV discordant individuals observed within this vaccine study. There is limited evidence that they contribute to differences observed, with a relative expansion of adaptive and contraction of canonical NK cells post vaccination, which is apparent in some but not all donors. However, the impact of HCMV on post-vaccination responses of NK cells was not limited to functional and metabolic outputs of adaptive NK cells. Rather, we saw similar trends of altered responses across all subsets of adaptive, canonical and even CD56^bright^ NK cells. In fact, canonical CD56^dim^ NK cells represented the greatest source of IFNγ post vaccination in HCMV+ individuals, and this subset was equally severely impaired post vaccination in HCMV+ donors. This strongly suggests that although FcεRγ1 and Syk can be used to identify particular subsets in HCMV+ individuals, the impact of HCMV is much more global and has wide-reaching effects on all NK cells. This concept is supported by an earlier study on CD56^bright^ NK cells in response to influenza vaccine^17^ and a comprehensive twin study in which HCMV discordancy was estimated to account for variation in 58% of a variety of immune parameters measured^51^. The global prevalence of HCMV is estimated at 86% and it substantially increases with age^52^. Given that HCMV can strongly impact ‘normal’ immune responses of healthy individuals, its impact on vaccinations needs to be understood, particularly where a vaccine for COVID-19 will be particularly important for older individuals. However, it is possible that for some vaccines, e.g. malaria, prior HCMV infection may actually confer an advantage to the host through improved NK cell-mediated antibody responses^53^.

## Methods

### Participants, ethics and regulatory approval

Healthy male and non-pregnant female volunteers aged 18–50 years were recruited for a Phase I dual-vaccine trial for HIV-1 and HCV (clinical trial NCT02362217)^19^. Approvals for the clinical trial study from which samples were used were as previously reported and all participants gave written informed consent^19^. Ethics for the current study was provided by the REC of St. James’s Hospital, Dublin 8 Tallaght Hospital/St. James’s Hospital Joint Research Ethics Committee (reference 2014/07/List 27) and for the healthy HCMV+ donors by the REC of School of Biochemistry and Immunology, Trinity College, Dublin 2 (reference BI-CG-311220).

### Study design and vaccinations

Cryopreserved PBMCs and serum were obtained from volunteers enrolled in PEACHI Phase I clinical trial^19^. Vaccines were manufactured in Compliance with Good Manufacturing Practice and stored at −80 °C until use and thawed prior to administration via intramuscular injection in the deltoid region of separate limbs^54,55^. Co-vacinees (*n* = 16 enrolled) were co-primed with ChAd3-NSmut and ChAdV63.HIVconsv (2.5 × 10^10^ and 5 × 10^10^ vp) and boosted with MVA-NSmut and MVA.HIVconsv at week 8 to maintain an equivalent total dose of MVA (2 × 10^8^ PFU). Samples for analysis included pre-vaccination (Day 0) and post-vaccination time points taken at Day 1 and weeks 1, 4, 8, 8 + 1 (Day 57), 9 and 12. Serum from *n* = 9–16 subjects was available for enzyme-linked immunosorbent assay (ELISA) analysis, while PBMCs from *n* = 4–12 co-vacinees were assessed for NK responses.

### Flow cytometry analysis of NK post-vaccination responses

Cryopreserved PBMCs (5 million/ml) were thawed, washed and rested for 2 h in RPMI-1640 (Gibco) at 37 °C, 5% CO_2_ for ex vivo analyses or stimulated with IL-12 (30 ng/ml, Miltenyi Biotec)/IL-15 (100 ng/ml, NCI Institute) at 37 °C for 18 h with GolgiPlug (BD Pharmingen) for the final 4 h. Cell surface and viability staining (Live Dead Near-IR; Invitrogen) was 20 min at 4 °C with antibodies: CD56, 1:100 (318318, BioLegend); CD71, 1:100 (334108, BioLegend); CD25, 1:100 (563701); CD3, 1:200 (558124); CD69, 1:50 (555748); CD57, 1:100 (563895) and CD98, 1:50 (556077) all from BD Biosciences. PBMCs were fixed using BD Cytofix/Cytoperm for 20 min before intracellular staining using anti-GZB (515405, BioLegend), anti-IFNγ (502536, BioLegend), anti-Syk (12-6696-42, Ebiosciences), anti-FCεrG1 (FCABS400F, Merck Millipore), anti-phospho S6 235/6 ribosomal protein (8520S, Cell Signaling Technologies) and anti-ATP5B subunit of ATP synthase (ab197649, Abcam) at 1:100 for 30 min. NK mitochondrial membrane potential was measured by incubating PBMCs with JC-1 (2 μM, Thermo Fisher) for 20 min at 37 °C. Oligomycin (2 μM, Sigma) and FCCP (2 μM, Sigma) were used as positive and negative controls, respectively. Mitochondrial mass was measured by incubating PBMCs for 30 min with Mitotracker CMXRos (100 nM, Thermo Fisher) at 37 °C, while NK nutrient uptake was assessed by incubating PBMCs for 1 h in 2-NBDG (50 μM, Thermo Fisher). At least 5000 viable singlet CD3^−^CD56^+^ lymphocyte events were acquired using a BD Fortessa LSR or BD Canto II cytometers. Data were analysed using FlowJo v10 (FlowJo, USA) and GraphPad Prism v7.0.

### Quantification of serum cytokine production, anti-CMV IgG and IFNγ ELISPOT assay

Cryopreserved serum was thawed on ice before quantification of anti-CMV IgG (Alpha Diagnostics), IL-2, IL-15 and IFNγ (BioLegend) by ELISA, according to the manufacturer’s instructions. HCMV IFNγ ELISPOT assays were performed on freshly isolated PBMCs against HCMV lysate^19^.

### Reporting summary

Further information on research design is available in the Nature Research Reporting Summary linked to this article.

## Supplementary information

Supplementary Information File

Reporting Summary

## Data Availability

The data that support the findings of this study are available from the corresponding author (C.M.G.) upon reasonable request. This study did not generate any unique code or datasets other than those included in this published article (and its Supplementary information files).
