# Supplementary material for: Specific human cytomegalovirus signature detected in NK cell metabolic changes post vaccination
Source: NPJ Vaccines. 2021 Sep 28;6:117. doi: 10.1038/s41541-021-00381-w (PMC8478984; doi:10.1038/s41541-021-00381-w)
Supplement: Supplementary file 1 — Supplementary Information File [file 41541_2021_381_MOESM1_ESM.pdf]

Supplementary Figure 1

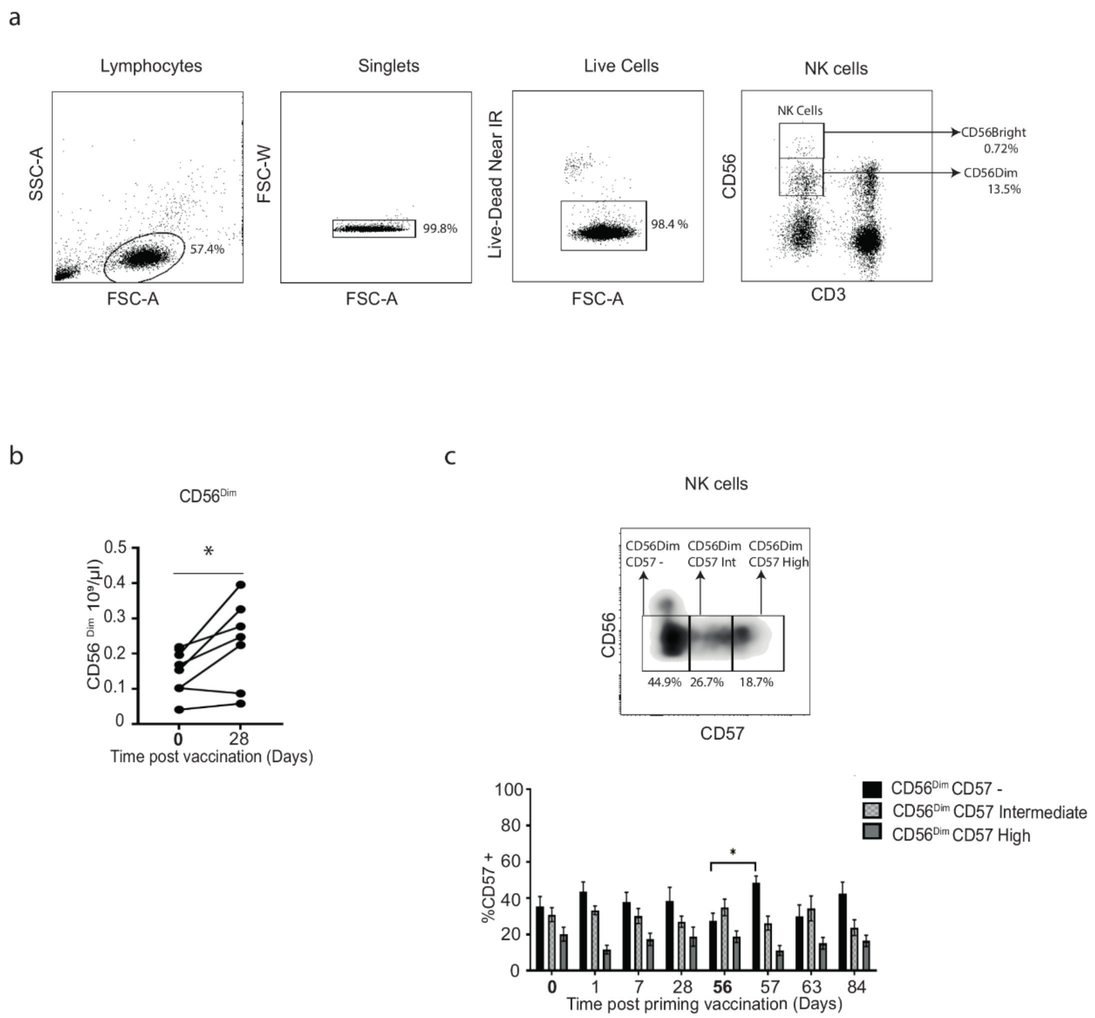

Supplementary Figure 1

(a) Example gating strategies for identification of NK cells in Supplemental Figure 1 and 3, CD56^{Bright} and CD56^{Dim} NK cells in Figures 1-5 and Supplemental Figures 1-7. Viable CD56+ NK cells were derived from singlet lymphocytes and gated on CD3- CD56+ populations. CD56^{Bright} and CD56^{Dim} subsets were defined based on intensity of CD56 expression. (b) *Ex vivo* Paired donor responses for CD56^{Dim} absolute numbers at D0-D28 (n=7). (c) Gating strategy for CD56Dim NK cells stratified by CD57 expression and Mean CD57 expression (n=6-8) by flow cytometry on CD3-CD56^{Dim} NK cells, error bars denote S.E.M. Samples were compared by (b) paired t-test and (c) two-way ANOVA with Sidek's post hoc test. *$p<0.05$.

Supplementary Figure 2

a

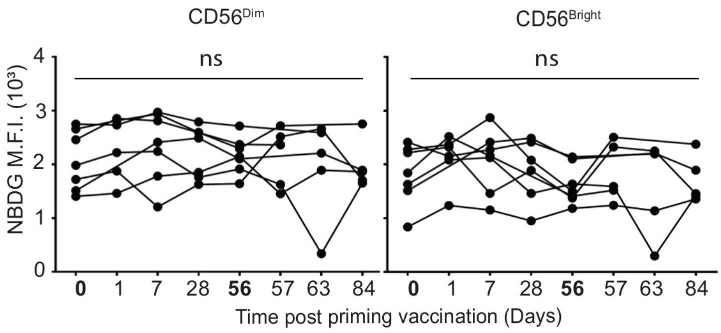

b

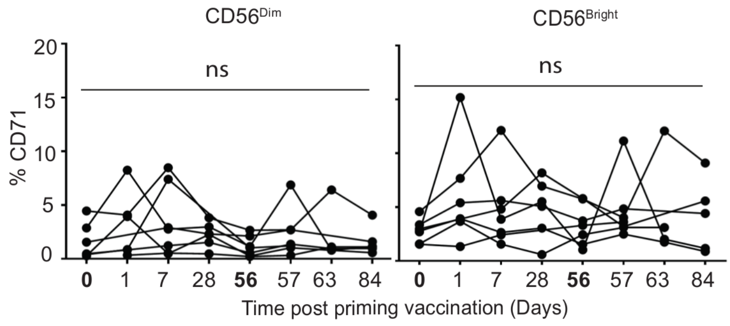

Supplementary Figure 2

NK nutrient uptake is not impacted by vaccination. $CD56^{Dim}$ and $CD56^{Bright}$ NK cells from cryopreserved PBMCs were analysed *ex vivo* by flow cytometry on for (a) 2-NBDG uptake (n=7 baseline, n=5-7 other timepoints) and (b) CD71 expression (n=6 baseline, n=5-7 other timepoints). (a, b) Samples were compared using one-way ANOVA with Sidek's post hoc test. ns= not significant.

Supplementary Figure 3

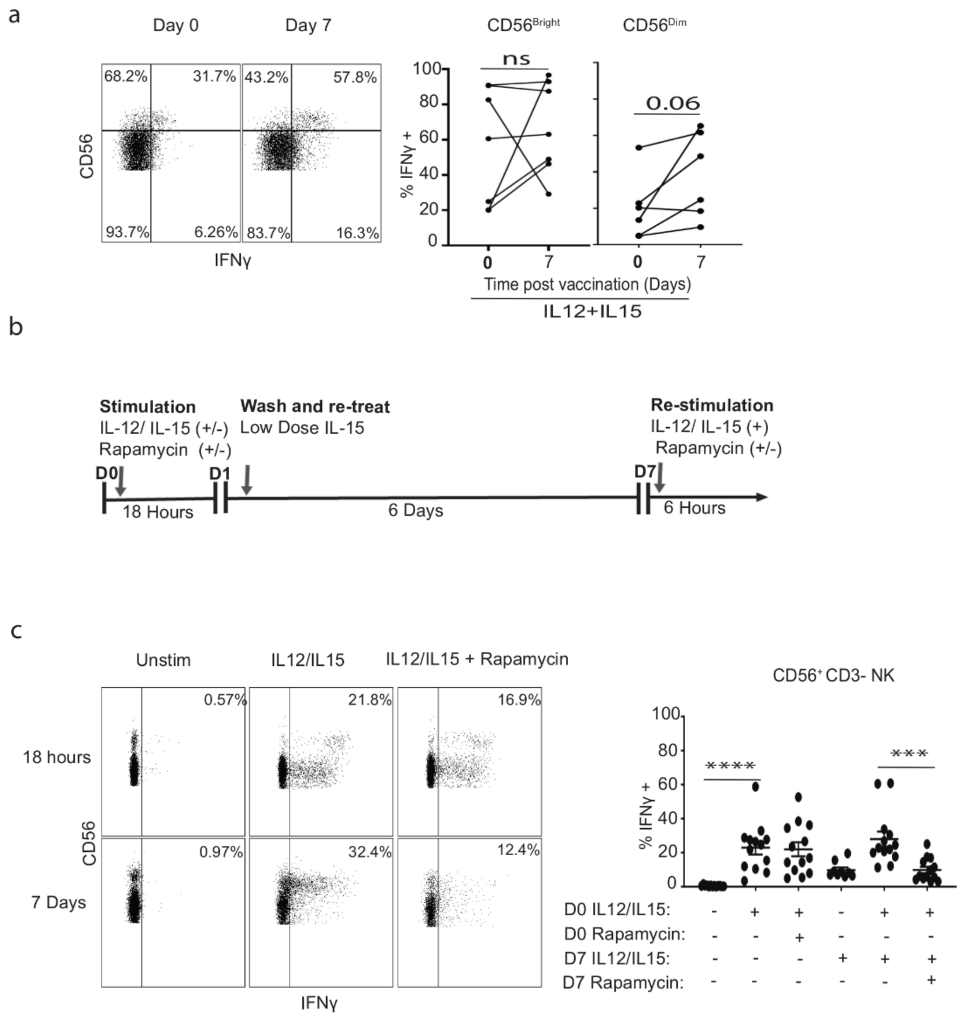

Supplementary Figure 3

Cytokine training of human NK cells *in vitro* is dependent on mTORC-1. (b)Sample Flow cytometry plots and paired IFNγ responses in CD56 ^{Dim} and CD56^{Bright} NK cells in response to 18 hours IL-12 (30ng) and IL-15 (100ng) stimulation at Day 0 and 7 after priming(n=6). (b) An *in vitro* model for generation of cytokine trained NK cells to examine the role of mTORC-1 in facilitating enhanced NK IFNγ production. PBMC were initially cultured in media or in the presence of IL-12 (30ng) and IL-15 (100ng) for 18 hours, with or without the mTORC-1 inhibitor rapamycin (20μM). Cultures were then washed extensively and maintained in the presence of IL-15 (1ng/ml) for 6 days to ensure NK survival. Cells were then re-stimulated for 6 hours with IL-12/IL-15 and treated with Golgi plug in conjunction with rapamycin, as indicated (c) Representative flow cytometry plot and summary of frequencies of IFNγ

producing NK cells after mTORC-1 inhibition during 7 day *in vitro* culture (n=13).) Samples were compared using paired t-test (a) and one-way ANOVA (c) ns=not significant, ***p<0.001, ****p<0.0001.

Supplementary Figure 4

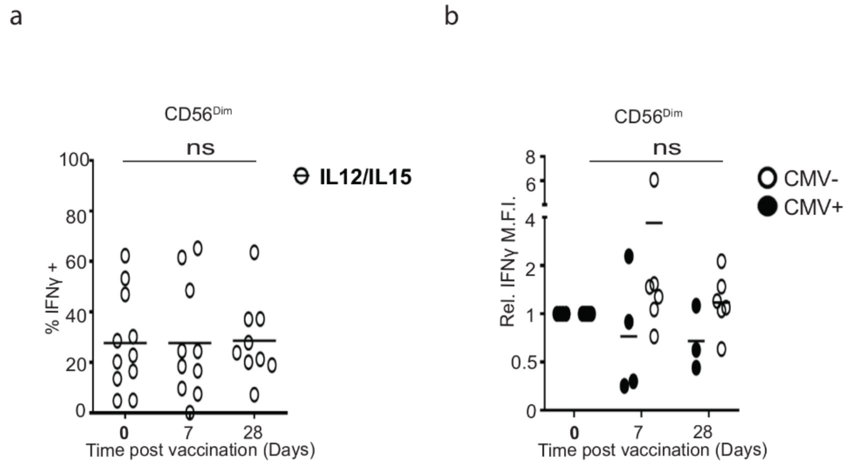

Supplementary Figure 4

(a) Summary graph of IL-12 (30ng) /IL-15 (100ng) stimulated IFN γ + frequencies in CD56Dim NKs independent of HCMV sero-status at Day 0 (n=11), Day 7 (n=10) and Day 28 (n=9). White circles: IL12/IL15.Line indicates the mean. (b) Summary graph of Relative Fluorescence intensity of IFNγ in CD56Dim NKs after vaccination normalized to baseline (Day 0) values in HCMV sero-discordant donors in response to IL-12/IL-15 stimulation (HCMV-, n=6. HCMV + D0,D7 n=4, D28 n=3).Dots indicate individual donor responses, while lines represent the mean. Samples were compared by (a) One-Way ANOVA and (b)Two-Way ANOVA with Sidek's post hoc test. ns= not significant

Supplementary Figure 5

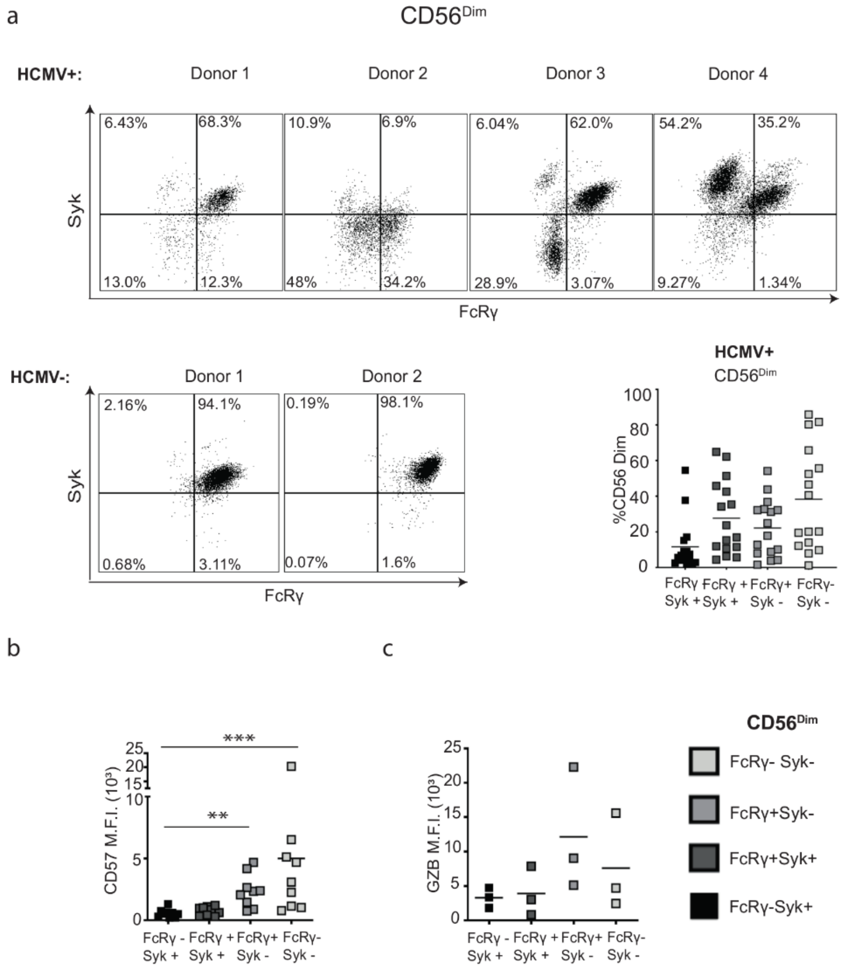

Supplementary Figure 5

Identification and phenotyping of Canonical (FcεRγ+ and Syk+) and Adaptive (FcεRγ- Syk-, FcεRγ- Syk+. FcεRγ+ Syk-) CD56 ^{Dim} NK cells. (a)Sample flow cytometry plots of CD56^{Dim} NK cells expressing FcεRγ and Syk signaling protein expression for two HCMV- and four HCMV+ donors, and a summary graph of *ex vivo* frequencies of canonical (both FcεRγ and Syk negative) and adaptive (all other combinations of FcεRγ and Syk) NK cells in HCMV+ donors (n=12). Summary of (b) CD57 (n=9) and (c) Granzyme B expression (n=3) in canonical and adaptive CD56^{Dim} NKs from HCMV+ donors. One Way ANOVA, Holm Sidaks test for multiple comparisons. **$p<0.01$, ***$p<0.001$

Supplementary Figure 6

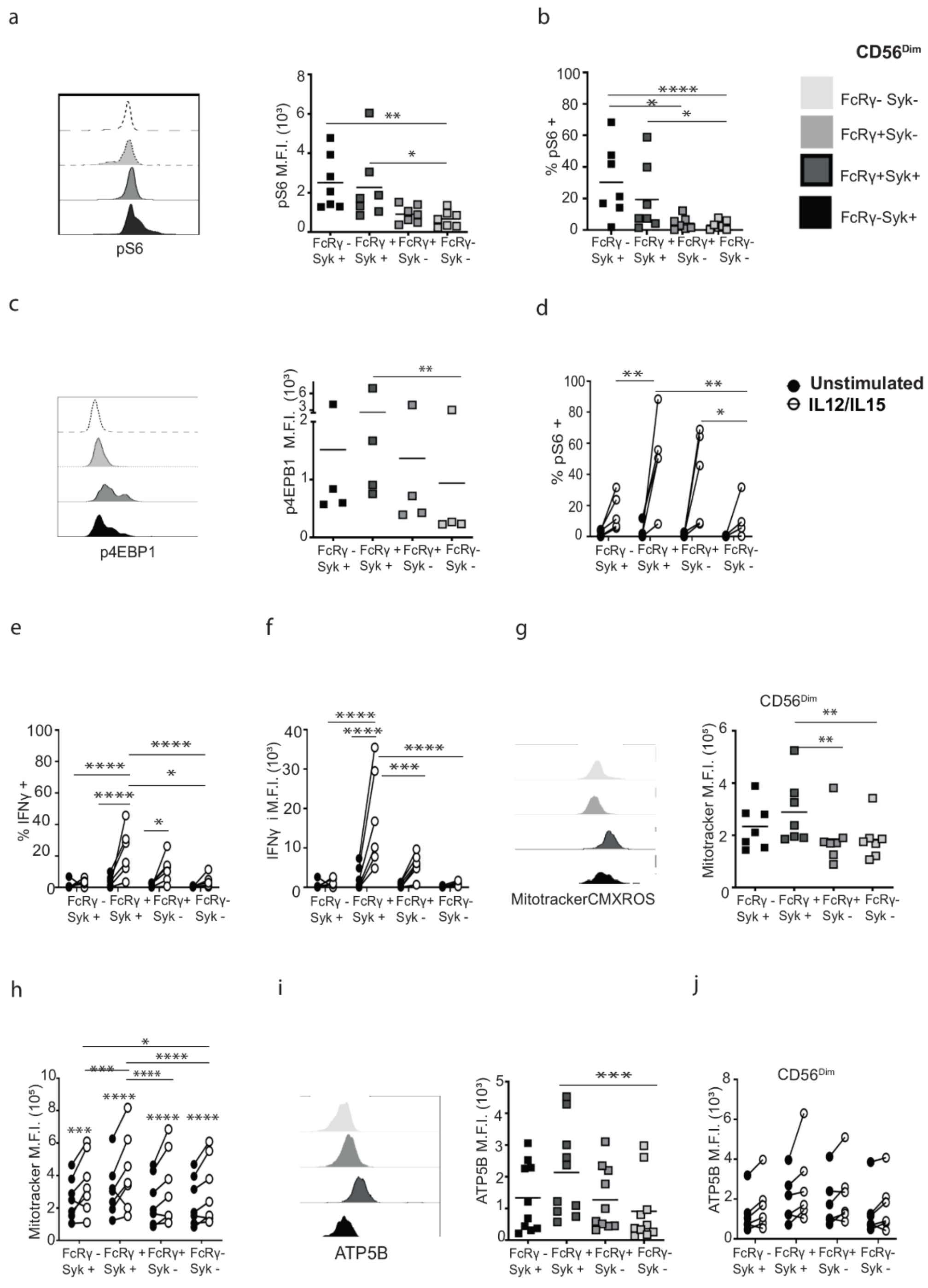

Supplementary Figure 6

Cytokine activated Canonical (FcεRγ+ and Syk+) CD56 ^{Dim} NK cells have higher IFNγ production and metabolic activity than Adaptive CD56 ^{Dim} NK cells. Ex *vivo* representative histogram and summary data for (a) pS6 M.F.I., (b) pS6+ % and (c) p4EBP-1 M.F.I. (A,B

n=7, C n=4). (D) Summary of pS6 expression (n=4) (e) frequency of IFNγ producing (n=6) and (f) integrated IFNγ M.F.I. responses in adaptive and canonical $CD56^{Dim}$ NK cell subsets in response to 18 hours IL-12 and IL-15 stimulation. Integrated M.F.I. was determined by multiplying % of IFNγ positive $CD56^{Dim}$ NK cells by M.F.I. of IFNγ positive NK cells. Black unstimulated, white IL-12/IL-15. n=6. (g) Representative histogram and summaries of Mitotracker CMX ROS (n=7) and (i) ATP5B M.F.I. *ex vivo* (n=10) and (h,j) in response to IL-12/IL-15 stimulation in canonical and adaptive NK subsets. Black unstimulated, white IL-12/IL-15. n=6. (a-c,g,i) Samples were compared by One-Way ANOVA. (d-f,h,j) Samples were compared by Two-Way ANOVA with Sidek's post hoc test. *p<0.05, **p<0.01, ***p<0.001, ****p<0.0001.

Supplementary Figure 7

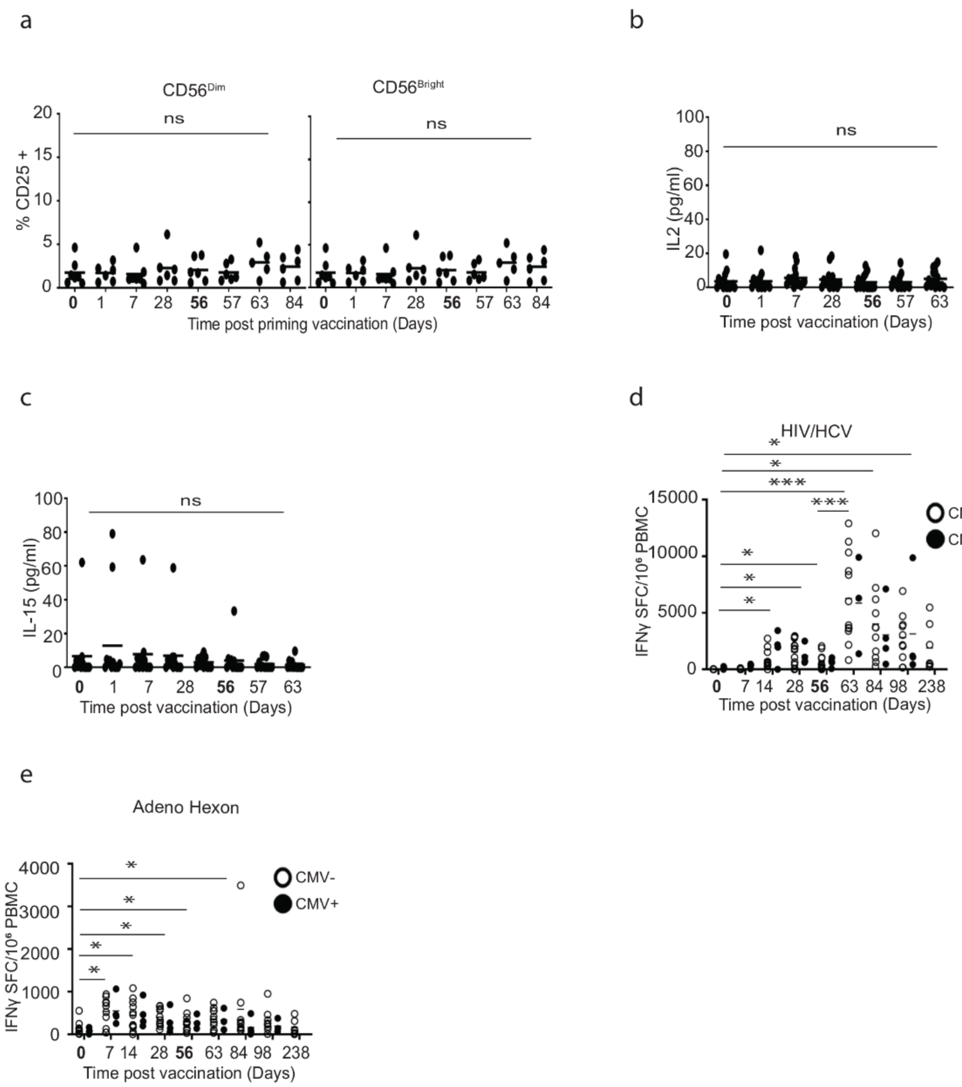

Supplementary Figure 7

(a) Summary graphs of CD25 expression on CD56^{Dim} and CD56^{Bright} NK cells measured by flow cytometry in cryopreserved PBMC at baseline and indicated time points post vaccination (Baseline, n=7. Other time points n=5-7). (b) Summary graphs of IL-2 and (c) IL-15 expression(n=12). (d) Cumulative responses to HCVNSmut and HIVconsv (n=4-12) and (e)Adeno Hexon (n=4-12) as determined by fresh *ex vivo* IFN-γ ELISPOT assays stratified by HCMV status. Black Dots indicate individual donor responses, while lines represent the mean.Black dots indicate HCMV seropositive donors, white dots indicate HCMV seronegative. Samples were compared by One-Way ANOVA (a-c) or Two-Way ANOVA (d-e) with Sidek's post hoc test. *p<0.05, ***p<0.001

PEACHI Consortium members:

Ciaran Bannan[2], Eleanor Barnes[6,11], Colm Bergin[2], Carly Bliss[6], Dorota Borys[7], Dominique Boutriau[7], Anthony Brown[6], Stefania Capone[8], Paola Cicconi[9], Landry Cochard[7], Annette von Delft[6], Lucy Dorrell[6,11], Ilaria Esposito[6], Marialuisa Esposito[8], Antonella Folgori[8], Clair Gardiner[1], Emma Ghaffari[6], Tomáš Hanke[9,10], Felicity Hartnell[6], Matthias Hoffmann[4,5], Jakub Kopycinski[6], Lan Lin[7], Shokouh Makvandi-Nejad[6], Federica Mori[8], Verity Nevin[6], Patrick Schmid[4], Leo Swadling[6], Frank Struyf[7], Bethany Turner[6], Ventzi Vassilev[7], Pietro Vernazza[4], Elena Woods[1]

Affiliations

1 School of Biochemistry and Immunology, Trinity Biomedical Sciences Institute, Trinity College, Dublin 2, Ireland

2 St James' Hospital, Dublin 8, Ireland

3 School of Pharmacy, Trinity College, Dublin 2, Ireland

4 Division of Infectious Diseases and Hospital Epidemiology, Kantonsspital St Gallen, St Gallen,
Switzerland

5 Department of Internal Medicine, Division of Infectious Diseases and Hospital Epidemiology,
Kantonsspital Olten, Olten, Switzerland

6 Nuffield Department of Medicine, University of Oxford, Oxford, UK

7 GlaxoSmithKline Vaccines, Brussels, Belgium

8 ReiThera s.r.l., Rome, Italy

9 The Jenner Institute, University of Oxford, Oxford, UK

10 Joint Research Center for Human Retrovirus Infection, Kumamoto University, Japan

11 Oxford NIHR Biomedical Research Centre, Oxford, UK
