# Supplementary material for: Specific human cytomegalovirus signature detected in NK cell metabolic changes post vaccination
Source: NPJ Vaccines. 2021 Sep 28;6:117. doi: 10.1038/s41541-021-00381-w (PMC8478984; doi:10.1038/s41541-021-00381-w)
Supplement: Supplementary file 2 — Reporting Summary [file 41541_2021_381_MOESM2_ESM.pdf]

nature research

Corresponding author(s): Clair Gardiner
Last updated by author(s): Jul 13, 2021

Reporting Summary

Nature Research wishes to improve the reproducibility of the work that we publish. This form provides structure for consistency and transparency in reporting. For further information on Nature Research policies, see our Editorial Policies and the Editorial Policy Checklist.

Statistics

For all statistical analyses, confirm that the following items are present in the figure legend, table legend, main text, or Methods section.

n/a	Confirmed	
☐	☒	The exact sample size (*n*) for each experimental group/condition, given as a discrete number and unit of measurement
☐	☒	A statement on whether measurements were taken from distinct samples or whether the same sample was measured repeatedly
☐	☒	The statistical test(s) used AND whether they are one- or two-sided<br>*Only common tests should be described solely by name; describe more complex techniques in the Methods section.*
☒	☐	A description of all covariates tested
☐	☒	A description of any assumptions or corrections, such as tests of normality and adjustment for multiple comparisons
☐	☒	A full description of the statistical parameters including central tendency (e.g. means) or other basic estimates (e.g. regression coefficient) AND variation (e.g. standard deviation) or associated estimates of uncertainty (e.g. confidence intervals)
☒	☐	For null hypothesis testing, the test statistic (e.g. *F*, *t*, *r*) with confidence intervals, effect sizes, degrees of freedom and *P* value noted<br>*Give P values as exact values whenever suitable.*
☒	☐	For Bayesian analysis, information on the choice of priors and Markov chain Monte Carlo settings
☒	☐	For hierarchical and complex designs, identification of the appropriate level for tests and full reporting of outcomes
☒	☐	Estimates of effect sizes (e.g. Cohen's *d*, Pearson's *r*), indicating how they were calculated

Our web collection on statistics for biologists contains articles on many of the points above.

Software and code

Policy information about availability of computer code

Data collection	FACS DIVA was used to collect Flow cytometry data.
Data analysis	Flowjo v10.6.1 and GraphPad Prism 9 were used to analyse data.

For manuscripts utilizing custom algorithms or software that are central to the research but not yet described in published literature, software must be made available to editors and reviewers. We strongly encourage code deposition in a community repository (e.g. GitHub). See the Nature Research guidelines for submitting code & software for further information.

Data

Policy information about availability of data

All manuscripts must include a data availability statement. This statement should provide the following information, where applicable:

- Accession codes, unique identifiers, or web links for publicly available datasets
- A list of figures that have associated raw data
- A description of any restrictions on data availability

The data that support these findings are available from the corresponding author (C.G.) upon reasonable request. This study did not generate any unique code or dataset other than those included in this published article (and its supplementary information files).

Field-specific reporting

Please select the one below that is the best fit for your research. If you are not sure, read the appropriate sections before making your selection.

☒ Life sciences ☐ Behavioural & social sciences ☐ Ecological, evolutionary & environmental sciences

For a reference copy of the document with all sections, see nature.com/documents/nr-reporting-summary-flat.pdf

Life sciences study design

All studies must disclose on these points even when the disclosure is negative.

Sample size	We have original power calculations and these vary depending on the assay. We have been working in this area for almost 20 years and use statistics appropriate to the data being presented. With human studies, our samples usually range between 5-20 for individual experimental data sets.
Data exclusions	We include all data. Humans are notoriously variable compared to mice (which are usually inbred). Capturing the diversity of the 'normal' response is critical for research to be relevant for human health.
Replication	We have internal replicates where appropriate e.g. ELISA. All the data presented are the independent biological replicates.
Randomization	Most of the samples are longitudinal and within a person. Randomisation was not relevant.
Blinding	not relevant to this study

Reporting for specific materials, systems and methods

We require information from authors about some types of materials, experimental systems and methods used in many studies. Here, indicate whether each material, system or method listed is relevant to your study. If you are not sure if a list item applies to your research, read the appropriate section before selecting a response.

Materials & experimental systems

n/a	Involved in the study	
☐	☒	Antibodies
☒	☐	Eukaryotic cell lines
☒	☐	Palaeontology and archaeology
☒	☐	Animals and other organisms
☐	☒	Human research participants
☒	☐	Clinical data
☒	☐	Dual use research of concern

Methods

n/a	Involved in the study	
☐	☐	ChIP-seq
☐	☒	Flow cytometry
☐	☐	MRI-based neuroimaging

Antibodies

Antibodies used	Antibodies: CD56 (NCAM16.2), CD71 (CY1G4) (Medical Supply Co); CD25 (M-A251), CD3 (UCHT1), CD69 (L78,), CD57 (NK-1), CD98 (UM7F8) (BD Pharmingen), anti-granzyme B (GB11), anti-IFNγ (B27), anti-Syk (4D10.1) all from BD Pharmingen, anti-FCεrG1 (Merck Millipore), anti-phospho S6 ribosomal protein (phospho-serine 235/6, Cell Signaling Technologies) and anti-ATP5B subunit of ATP Synthase (3D5, Abcam).
Validation	These are all antibodies that are validated by the manufacturer on human PBMC and widely used and widely published upon in the field including the following studies: Schlums et al 2016 PMID: 25786176. Keating et al, 2016 , PMID: 26873994. Slattery et al 2021, PMID: 33568351.

Human research participants

Policy information about studies involving human research participants

Population characteristics	Healthy adult volunteers (aged 18-50) enrolled in PEACHI Phase I clinical trial, as described in Hartnell et al, 2018 PMID 30713538.<br><br>Peripheral blood was obtained from unvaccinated donors (age 22-50) working in Trinity Biomedical Science Institute, Dublin Ireland.
Recruitment	Recruited by collaborators in Oxford as part of Peach I clinical trial as above. Unvaccinated donors were recruited in TBSI with no selection bias other than to be HCMV positive.

Ethics oversight	Approvals for the clinical trial study from which samples were used was as previously reported (19). Ethics for this study was provided by the REC of St. James's Hospital, Dublin 8 Tallaght Hospital / St James's Hospital Joint Research Ethics Committee (reference 2014/07/List 27) and for the healthy HCMV+ donors by the REC of School of Biochemistry and Immunology, Trinity College, Dublin 2 (reference BI-CG-311220).

Note that full information on the approval of the study protocol must also be provided in the manuscript.

ChIP-seq

Data deposition

☐ Confirm that both raw and final processed data have been deposited in a public database such as GEO.

☐ Confirm that you have deposited or provided access to graph files (e.g. BED files) for the called peaks.

Data access links<br>*May remain private before publication.*	*For "Initial submission" or "Revised version" documents, provide reviewer access links. For your "Final submission" document, provide a link to the deposited data.*
Files in database submission	*Provide a list of all files available in the database submission.*
Genome browser session<br>(e.g. UCSC)	*Provide a link to an anonymized genome browser session for "Initial submission" and "Revised version" documents only, to enable peer review. Write "no longer applicable" for "Final submission" documents.*

Methodology

Replicates	*Describe the experimental replicates, specifying number, type and replicate agreement.*
Sequencing depth	*Describe the sequencing depth for each experiment, providing the total number of reads, uniquely mapped reads, length of reads and whether they were paired- or single-end.*
Antibodies	*Describe the antibodies used for the ChIP-seq experiments; as applicable, provide supplier name, catalog number, clone name, and lot number.*
Peak calling parameters	*Specify the command line program and parameters used for read mapping and peak calling, including the ChIP, control and index files used.*
Data quality	*Describe the methods used to ensure data quality in full detail, including how many peaks are at FDR 5% and above 5-fold enrichment.*
Software	*Describe the software used to collect and analyze the ChIP-seq data. For custom code that has been deposited into a community repository, provide accession details.*

Flow Cytometry

Plots

Confirm that:

☐ The axis labels state the marker and fluorochrome used (e.g. CD4-FITC).

☒ The axis scales are clearly visible. Include numbers along axes only for bottom left plot of group (a 'group' is an analysis of identical markers).

☐ All plots are contour plots with outliers or pseudocolor plots.

☒ A numerical value for number of cells or percentage (with statistics) is provided.

Methodology

Sample preparation	For studies on unvaccinated HCMV+ donors human plasma was isolated by centrifugation, frozen and screened for HCMV anti-pp65 IgG. Peripheral blood mononuclear cells were isolated from blood(40ml) using Lymphoprep . Cryopreserved PBMC from the PEACHI 04 Phase I Clinical Trial (Hartnell et al, 2018 PMID: 30713538) were thawed, washed and rested for 2 hours in RPMI-1640 (GIBCO) at 37°C, 5% CO2 for ex vivo analyses or for cytokine production analysis cells were stimulated with IL12 (30ng/ml, Miltenyi Biotec)/ IL15 (100ng/ml, NCI Institute) at 37°C for 18 hours with GolgiPlug (BD Pharmingen) for the final 4 hours. Cells treated for 18 hours with cytokine plus Rapamycin (20nM) were included as a negative control for pS6 staining when cell numbers allowed.<br><br>For flow cytometry staining: Cells were stained with NIR-LIVE/ DEAD (Life Technologies) for 10 minutes then washed with PBS, 5% FBS, twice. Cells were then stained with antibodies diluted in PBS, FBS 5% for 20 minutes at 4C. Cells were then washed twice before data collection. For intracellular staining, following surface staining, cells were fixed and permeabilized (20 minutes) (BD Cytofix/cytoperm). Fixed cells were then incubated with antibodies for 30 minutes at 4C. Cells were then washed twice before data collection.<br><br>For metabolic flow cytometry analysis: Cells were incubated with 2-NBDG (1 hour) (ThermoFisher) or Mitotracker CMXROS (30 minutes) (Invitrogen) at 37°C before flow cytometry staining as described above.

Cryopreserved serum or plasma was thawed on ice before ELISA analysis for cytokines IFN-y, IL-2, IL-12 and IL-15 (Biolegend), as well as HCMV anti-pp65 IgG (Alpha Diagnostics).

Instrument

BD Fortessa LSR or BD Canto II

Software

BD FACSDIVA and FLOWJO (V10) software were used for data collection and data analysis respectively.

Cell population abundance

For flow cytometry analysis a minimum of 4000-5000 NK cells were recorded.

Gating strategy

Global cell population is gated on FSC-A/SSC-A. Single cells are gated on FSC-A/FSC-W. Live cells - using NIR LIVE/DEAD are gated. The dead population is determined using cells pretreated with ethanol to induce cell death as a positive control. NK cells were gated as CD56+/CD3-.
The IFNg+ and p-S6+ populations were determine using the corresponding negative population in non-stimulated cells or fluorescence minus one controls.

☒ Tick this box to confirm that a figure exemplifying the gating strategy is provided in the Supplementary Information.

Magnetic resonance imaging

Experimental design

Design type

Indicate task or resting state; event-related or block design.

Design specifications

Specify the number of blocks, trials or experimental units per session and/or subject, and specify the length of each trial or block (if trials are blocked) and interval between trials.

Behavioral performance measures

State number and/or type of variables recorded (e.g. correct button press, response time) and what statistics were used to establish that the subjects were performing the task as expected (e.g. mean, range, and/or standard deviation across subjects).

Acquisition

Imaging type(s)

Specify: functional, structural, diffusion, perfusion.

Field strength

Specify in Tesla

Sequence & imaging parameters

Specify the pulse sequence type (gradient echo, spin echo, etc.), imaging type (EPI, spiral, etc.), field of view, matrix size, slice thickness, orientation and TE/TR/flip angle.

Area of acquisition

State whether a whole brain scan was used OR define the area of acquisition, describing how the region was determined.

Diffusion MRI ☐ Used ☐ Not used

Preprocessing

Preprocessing software

Provide detail on software version and revision number and on specific parameters (model/functions, brain extraction, segmentation, smoothing kernel size, etc.).

Normalization

If data were normalized/standardized, describe the approach(es): specify linear or non-linear and define image types used for transformation OR indicate that data were not normalized and explain rationale for lack of normalization.

Normalization template

Describe the template used for normalization/transformation, specifying subject space or group standardized space (e.g. original Talairach, MNI305, ICBM152) OR indicate that the data were not normalized.

Noise and artifact removal

Describe your procedure(s) for artifact and structured noise removal, specifying motion parameters, tissue signals and physiological signals (heart rate, respiration).

Volume censoring

Define your software and/or method and criteria for volume censoring, and state the extent of such censoring.

Statistical modeling & inference

Model type and settings

Specify type (mass univariate, multivariate, RSA, predictive, etc.) and describe essential details of the model at the first and second levels (e.g. fixed, random or mixed effects; drift or auto-correlation).

Effect(s) tested

Define precise effect in terms of the task or stimulus conditions instead of psychological concepts and indicate whether ANOVA or factorial designs were used.

Specify type of analysis: ☐ Whole brain ☐ ROI-based ☐ Both

Statistic type for inference
(See Eklund et al. 2016)

Specify voxel-wise or cluster-wise and report all relevant parameters for cluster-wise methods.

Correction | *Describe the type of correction and how it is obtained for multiple comparisons (e.g. FWE, FDR, permutation or Monte Carlo).*

Models & analysis

n/a	Involved in the study
☐	☐ Functional and/or effective connectivity
☐	☐ Graph analysis
☐	☐ Multivariate modeling or predictive analysis

Functional and/or effective connectivity | *Report the measures of dependence used and the model details (e.g. Pearson correlation, partial correlation, mutual information).*

Graph analysis | *Report the dependent variable and connectivity measure, specifying weighted graph or binarized graph, subject- or group-level, and the global and/or node summaries used (e.g. clustering coefficient, efficiency, etc.).*

Multivariate modeling and predictive analysis | *Specify independent variables, features extraction and dimension reduction, model, training and evaluation metrics.*
